# Emotional intelligence and depression, anxiety, and stress symptoms among clinical nurses: a cross-sectional network analysis

**DOI:** 10.3389/fpsyg.2026.1894001

**Published:** 2026-09-30

**Authors:** Man Wei, Wei Lu, Chengxin Bai, Xuqing Fan, Dongmei Wu

**Affiliations:** 1 College of Nursing, Chengdu University of Traditional Chinese Medicine, Chengdu, China; 2 Department of Nursing, Sichuan Provincial People's Hospital, School of Medicine, University of Electronic Science and Technology of China, Chengdu, China

**Keywords:** clinical nurses, emotional intelligence, depression, anxiety, stress, network analysis

## Abstract

**Background:**

Clinical nurses are at risk of depression, anxiety, and stress because of high work demands and sustained emotional labor. Emotional intelligence may be related to these negative affective symptoms, but the symptom-level links between emotional intelligence dimensions and specific symptoms remain unclear.

**Methods:**

This cross-sectional study recruited clinical nurses from hospitals in Southwest China between January 2021 and March 2022 using convenience sampling. A total of 704 clinical nurses were included. Emotional intelligence was assessed using the Wong and Law Emotional Intelligence Scale (WLEIS), and depression, anxiety, and stress symptoms were assessed using the simplified Chinese version of the Depression Anxiety Stress Scales-21 (DASS-21). A Gaussian graphical model was estimated using EBICglasso. Expected influence and bridge expected influence were used to identify central and bridge nodes. Network stability, exploratory subgroup differences by patient aggression experience and psychiatric versus non-psychiatric nursing status, and robustness in sensitivity analyses separately adjusting for age and years of work experience were examined.

**Results:**

The three largest absolute cross-community edge weights were ROE (“Regulation of emotion”)-DASS_18 (“I felt that I was rather touchy”), UOE (“Use of emotion”)-DASS_16 (“I was unable to become enthusiastic about anything”), and ROE-DASS_09 (“I was worried about situations in which I might panic and make a fool of myself”). Among the 21 DASS-21 symptom nodes, DASS_11 (“I found myself getting agitated”) had the highest expected influence, whereas among the four WLEIS dimension nodes, ROE had the largest absolute bridge expected influence. The main cross-community edges, central nodes, and bridge nodes remained largely stable after separate adjustment for age and years of work experience.

**Conclusion:**

Associations between emotional intelligence and negative affective symptoms were concentrated in specific nodes. DASS_11 may be a key central symptom, and ROE may be an important bridge linking emotional intelligence with depression, anxiety, and stress. These findings warrant further investigation in longitudinal and intervention studies.

## 1 Introduction

Clinical nurses are a core component of the healthcare workforce and play an essential role in patient safety and care quality. However, nursing work often involves heavy workload, rotating shifts, complex care demands, and sustained emotional labor, which increase the risk of depression, anxiety, stress, and other adverse mental health outcomes (Dræbel et al., 2026; Li et al., 2023). Previous studies have shown that these psychological problems are common among clinical nurses (Kunz et al., 2021; Salari et al., 2020), with prevalence rates often exceeding those in the general population; global evidence suggests that up to one-third of nurses may experience psychological distress (Al Maqbali et al., 2021; Ślusarska et al., 2022). Negative affective symptoms in nurses not only affect their mental health and quality of life, but are also associated with burnout, turnover intention, workforce loss, and poorer care quality (Buitron Saavedra et al., 2025; Habibpour et al., 2026).

Emotional intelligence has been increasingly recognized as a psychological resource that may be relevant to nurses’ mental health. It refers to the ability to perceive, understand, use, and regulate emotions in oneself and others, and to apply emotional information to guide thinking and behavior (Freshman and Rubino, 2002; Salovey and Mayer, 1990). In clinical nursing, these abilities may be particularly important because nurses are required to respond to patients’ suffering, manage emotionally charged interactions, and maintain professional functioning under sustained work pressure. Gross’s process model of emotion regulation provides a framework for understanding how emotional intelligence may be related to symptoms of depression, anxiety, and stress. According to this model, individuals attend to and appraise emotionally relevant situations; emotional responses then develop, and regulation can occur at different stages of this process (Gross, 1998). Individuals may draw on their emotional abilities throughout these stages to process emotional information and regulate emotional responses. Consistent with this perspective, emotional intelligence is closely related to the selection and use of emotion-regulation strategies (Peña-Sarrionandia et al., 2015). In nursing contexts characterized by sustained work pressure and emotional labor, nurses repeatedly encounter emotionally demanding situations in which these abilities may be particularly relevant. The emotional intelligence dimensions examined in this study reflect capacities involved in emotion processing and regulation, whereas the items of the 21-item Depression Anxiety Stress Scales (DASS-21) capture specific manifestations of depression, anxiety, and stress (Lovibond and Lovibond, 1995). Their theoretical linkage within the broader processes of emotion generation and regulation provides a substantive basis for examining the conditional associations between emotional intelligence dimensions and specific negative affective symptoms within the same network. Previous studies have shown that higher emotional intelligence is associated with better psychological adjustment, lower stress, and fewer symptoms of depression, anxiety, and stress in healthcare professionals (Abualruz et al., 2024; Kuday and Tekin, 2026; Saikia et al., 2024; Xie et al., 2021). Together, this theoretical and empirical evidence supports further examination of the associations between different dimensions of emotional intelligence and specific symptoms of depression, anxiety, and stress among clinical nurses.

Although previous studies have shown that emotional intelligence is negatively associated with poor mental health outcomes, several gaps remain (Xu et al., 2026). Most studies have used correlation, regression, or total-score analyses to examine the relationship between emotional intelligence and depression, anxiety, and stress (Abualruz et al., 2024; Chen et al., 2024; Shi et al., 2022; Sun et al., 2021; Yu et al., 2023). These methods can describe overall associations, but they provide limited information about how specific dimensions of emotional intelligence are related to individual symptoms. Traditional latent variable approaches also tend to treat symptoms as indicators of broader psychological constructs, which may overlook the links among individual symptoms (Borsboom, 2017; Borsboom and Cramer, 2013; Hofmann et al., 2016). Network analysis provides a useful way to examine these symptom-level relationships. In a symptom network, psychological variables or symptom items are treated as nodes, and the associations between them are represented as edges (Epskamp et al., 2018; Robinaugh et al., 2020). Compared with analyses based only on total or subscale scores, network analysis can show how specific symptoms and psychological dimensions are connected. The 16 items of the Wong and Law Emotional Intelligence Scale (WLEIS) are organized around four theoretically defined dimensions of emotional intelligence; therefore, the four dimension scores were used as emotional intelligence nodes in this study (Wong and Law, 2002). In contrast, although the DASS-21 comprises three dimensions of depression, anxiety, and stress, each dimension includes several distinct symptom manifestations (Lovibond and Lovibond, 1995). Accordingly, all DASS-21 items were retained as symptom nodes to avoid obscuring symptom-level differences through aggregation and to examine the conditional associations between different emotional intelligence dimensions and specific negative affective symptoms. Network analysis can also help identify relatively important nodes through centrality and predictability indices (Yuan et al., 2022). In addition, bridge centrality can be used to identify nodes that connect emotional intelligence with symptoms of depression, anxiety, and stress.

Job Demands–Resources theory provides an overarching framework for understanding the relationships of occupational stress exposure and nursing work contexts with nurses’ psychological states. According to this theory, job characteristics that require sustained physical, cognitive, or emotional effort constitute job demands, and persistent or excessive job demands may increase psychological strain and the risk of adverse psychological states (Bakker and Demerouti, 2024). Patient aggression represents a specific interpersonal and emotional job demand. Previous studies have found that workplace violence is associated with emotional intelligence among healthcare workers and with an increased subsequent risk of depression and anxiety among nurses (Cao et al., 2022; Zhang et al., 2025). Psychiatric nursing also involves specific interpersonal interactions and emotional job demands, with related occupational stressors being associated with emotional intelligence and psychological distress among psychiatric nurses (Liu et al., 2023; Qiu et al., 2025). This evidence provides a basis for further exploring the network patterns of emotional intelligence and negative affective symptoms across patient aggression experiences and nursing work contexts. In addition, age and years of work experience may represent important individual and occupational background factors when examining the relationship between emotional intelligence and negative affective symptoms. Age and years of work experience reflect an individual’s developmental stage and accumulated professional nursing experience, respectively, and may be associated with corresponding background differences in emotional intelligence dimensions and negative affective symptoms. Recent research has found that nurses’ emotional intelligence is associated with both age and years of work experience (Mehralian et al., 2024); age is also associated with anxiety and stress symptoms among nurses (Mohamed et al., 2025), while years of work experience is related to the risk of developing stress symptoms (Hu et al., 2024). These findings suggest that age and professional experience should be considered when assessing the robustness of the main network findings.

Based on this rationale, the present study aimed to use network analysis to examine the conditional associations between dimensions of emotional intelligence and specific symptoms of depression, anxiety, and stress among clinical nurses, and to identify central and bridge nodes. As an exploratory analysis, we also examined whether network patterns differed between nurses with and without patient aggression experience. Given the relatively high proportion of psychiatric nurses in the sample, we further conducted a *post hoc* exploratory network comparison between psychiatric and non-psychiatric nurses to examine whether network patterns differed across nursing work contexts. In addition, separate sensitivity analyses were conducted for age and years of work experience to assess the robustness of the main network findings.

## 2 Methods

### 2.1 Study design and participants

This cross-sectional study was reported in accordance with the Strengthening the Reporting of Observational Studies in Epidemiology (STROBE) guidelines. Data were collected from clinical nurses in Southwest China, mainly in Chengdu and Kunming, between January 2021 and March 2022. Participants were recruited through hospital nursing departments using convenience sampling. The network comprised 25 nodes. Sample size requirements for network analysis depend on several factors, including the number of nodes, network structure, and analytic aims (Epskamp and Fried, 2018). Subsequent bootstrap analyses showed acceptable precision of the edge-weight estimates and stability of the centrality indices (Epskamp et al., 2018). Considering the number of nodes and the bootstrap results, the available sample was considered adequate for the present network analysis.

Inclusion criteria were as follows: (1) holding a valid nursing license; (2) providing informed consent; and (3) having at least 6 months of clinical experience. Exclusion criteria included: (1) being a nursing intern; (2) being on leave or receiving further training during the survey period; and (3) self-reporting a history of psychiatric or psychological disorders during the initial eligibility screening.

A total of 756 nurses started the survey. Of these, 709 completed the questionnaire and 47 did not complete it, yielding a completion rate of 93.8%. Among the 709 completed questionnaires, five respondents who did not hold a valid nursing license were excluded. The final analytic sample included 704 clinical nurses. In the final analytic sample, no data were missing for any study variable, so no imputation was performed; no duplicate responses or implausible values were identified during data cleaning. A participant flow diagram is provided in Supplementary Figure S1. This study was approved by the Ethics Committee of Chengdu Fourth Hospital, China (approval No. [2019] Ethics No. 01). The study was registered in the Chinese Clinical Trial Registry [ChiCTR1900020715]. All procedures were conducted in accordance with the Declaration of Helsinki. All participants provided informed consent before completing the survey.

### 2.2 Measures

#### 2.2.1 Demographic information

Demographic and occupational characteristics were collected using a self-designed questionnaire developed by the research team. The questionnaire included sex, age, years of work experience, marital status, educational attainment, professional title, department, hospital grade, hospital specialty, and patient aggression experience.

#### 2.2.2 Emotional intelligence scale

The Wong and Law Emotional Intelligence Scale (WLEIS), developed by Wong and Law in 2002 (Wong and Law, 2002), comprises 16 items grouped into four dimensions: self-emotion appraisal (SEA), others’ emotion appraisal (OEA), use of emotion (UOE), and regulation of emotion (ROE). Responses are scored on a 7-point Likert scale from 1 (strongly disagree) to 7 (strongly agree), with higher scores reflecting greater emotional intelligence. The original study supported the four-factor structure and internal consistency of the scale, and subsequent studies have further supported its psychometric properties in Chinese samples (Kong, 2017; Shi and Wang, 2007; Wong and Law, 2002). In the present study, the Cronbach’s α coefficient was 0.945 for the total scale, while the corresponding values for SEA, OEA, UOE, and ROE were 0.855, 0.856, 0.876, and 0.912, respectively.

#### 2.2.3 Simplified Chinese version of the Depression Anxiety Stress Scales-21

Negative affective symptoms were assessed using the simplified Chinese version of the Depression Anxiety Stress Scales-21 (DASS-21) (Taouk et al., 2001). The DASS-21 was originally developed by Lovibond and Lovibond to assess symptoms of depression, anxiety, and stress over the past week (Lovibond and Lovibond, 1995). The simplified Chinese version used in this study has been validated for use in mainland Chinese populations. Previous studies have supported the cross-cultural validity of the Chinese DASS-21 in mainland Chinese samples and its reliability and validity among Chinese hospital workers (Jiang et al., 2020; Wang et al., 2016). The scale contains 21 items, with seven items for each subscale: depression, anxiety, and stress. Each item is rated on a 4-point scale from 0 to 3. For descriptive and subscale-level analyses, scores were calculated by summing the seven items of each subscale and multiplying the subtotal by 2, with higher scores indicating more severe symptoms. The DASS-21 was used as a screening measure rather than a diagnostic instrument. In the present study, Cronbach’s α was 0.945 for the total scale, and the values for the depression, anxiety, and stress subscales were 0.863, 0.850, and 0.858, respectively.

### 2.3 Data collection

Data collection was conducted through an anonymous online survey. Before the survey, the nursing departments of the participating hospitals were contacted, and data collectors received unified training. Eligible nurses were then invited to complete the questionnaire online via a survey link distributed by the data collectors. The numbers of nurses who received the survey link and those who declined participation were not recorded. The introductory page explained the study purpose, procedures, potential risks and benefits. It informed participants that all data would be collected anonymously and that they could withdraw at any time before submission. To reduce the likelihood of duplicate submissions from the same IP address, the survey platform restricted submissions to one per IP address.

### 2.4 Statistical analysis

Descriptive statistics, correlation analysis, and reliability testing were conducted using IBM SPSS Statistics 26.0. Categorical variables were summarized as frequencies and percentages, and continuous variables were summarized as means and standard deviations. Pearson correlation analysis was used to examine bivariate associations among the main study variables. Network analyses were performed in R version 4.5.2.

#### 2.4.1 Network estimation and visualization

A Gaussian graphical model comprising 25 nodes was estimated, including four WLEIS dimension scores and 21 DASS-21 symptom items. The WLEIS was represented at the dimension level to reflect its established four-dimensional structure, whereas the DASS-21 items were retained individually to preserve symptom-level information. To account for differences in measurement level, the correlation matrix was computed using cor_auto, and the network structure was subsequently estimated using the EBICglasso procedure (Isvoranu and Epskamp, 2023). This procedure applies graphical least absolute shrinkage and selection operator regularization and selects the optimal network using the Extended Bayesian Information Criterion (Epskamp and Fried, 2018; Friedman et al., 2008). The EBIC tuning parameter was set to 0.5. Edges represented regularized partial correlations between two nodes after conditioning on all other nodes in the network. Network visualization was conducted using the qgraph package.

#### 2.4.2 Centrality estimation

Expected influence (EI) was used as the primary centrality index because the network included both positive and negative edges (Robinaugh et al., 2016). Closeness and betweenness were also estimated but were presented only as supplementary indices because they may be unstable in psychological networks (Epskamp et al., 2018). Node predictability was estimated using the R package mgm. Consistent with the Gaussian graphical model, predictability was expressed as R^2^ for all nodes, indicating the proportion of variance in each node explained by its neighboring nodes (Haslbeck and Waldorp, 2018). Bridge expected influence (BEI) was calculated using the networktools package to identify nodes connecting the emotional intelligence community and the depression, anxiety, and stress symptom community (Jones et al., 2021). The absolute magnitude of BEI was used to describe the prominence of cross-community connectivity, while its sign indicated the overall direction of the cross-community associations.

#### 2.4.3 Network accuracy and stability

The accuracy of edge weights and the stability of centrality indices were assessed using the R package bootnet. Edge-weight accuracy was evaluated using 95% bootstrap confidence intervals (CIs) based on 1,000 nonparametric bootstrap samples. Centrality stability was assessed using a case-dropping bootstrap procedure and the correlation stability coefficient (CS-coefficient). Correlation stability coefficients above 0.50 were considered to indicate good stability, whereas values below 0.25 were interpreted with caution (Epskamp et al., 2018).

#### 2.4.4 Network comparison

As an exploratory analysis, participants were divided into nurses with no patient aggression experience and those with any patient aggression experience to examine whether patient aggression experience was associated with differences in the network linking emotional intelligence with negative affective symptoms. This dichotomization was used because the sample sizes across the original categories were markedly unequal and did not support reliable separate network estimation by aggression type or severity. The two networks were compared using the Network Comparison Test (NCT) in R (van Borkulo et al., 2023). Both subgroup networks were estimated using the same cor_auto correlation specification and EBICglasso procedure as the primary network. Differences in overall network structure, global strength, and individual edge weights were examined using 1,000 permutations. The *p* values for edge-level comparisons were adjusted using the Benjamini–Hochberg procedure (Benjamini and Hochberg, 1995) to control the false discovery rate (FDR). Given the marked imbalance in subgroup sizes, the results were interpreted cautiously, particularly because the ability to detect subtle between-group differences may have been limited.

Participants were classified as psychiatric nurses or non-psychiatric nurses for the *post hoc* exploratory network comparison. The two networks were compared using the Network Comparison Test (NCT) in terms of overall network structure, global strength, and individual edge weights. The network estimation procedure and parameter settings were consistent with those used in the primary analysis. The test was based on 1,000 permutations, and *p* values for individual edge comparisons were adjusted using the Benjamini–Hochberg procedure to control the FDR.

#### 2.4.5 Sensitivity analyses

To examine the robustness of the main network findings, two sensitivity analyses were conducted by separately adjusting for age and years of work experience. In each analysis, every WLEIS dimension and DASS-21 item was regressed on the corresponding covariate, and the resulting residuals were used to re-estimate the network using the same estimation procedure and parameter settings as the primary network. The adjusted and unadjusted networks were then compared in terms of edge weights, key cross-community edges, centrality indices, and bridge indices. Case-dropping bootstrap analyses were also conducted to assess the stability of the centrality and bridge indices in both adjusted networks.

## 3 Results

### 3.1 Sample characteristics

A total of 704 nurses were included in the final analysis. The mean age of the participants was 31.8 ± 7.4 years, and the mean length of work experience was 10.7 ± 8.1 years. Most participants were female (90.6%), married (67.9%), held a bachelor’s degree (56.5%), and had the professional title of nurse (52.0%). Regarding hospital grade, 598 nurses (84.9%) worked in tertiary grade A hospitals, 90 (12.8%) in secondary grade A hospitals, and 16 (2.3%) in tertiary grade B hospitals. Regarding hospital specialty, 331 nurses (47.0%) worked in psychiatric specialty hospitals and 373 (53.0%) in non-psychiatric hospitals. In this sample, all psychiatric nurses worked in psychiatric specialty hospitals. In total, 130 nurses (18.5%) reported no patient aggression experience, whereas 574 nurses (81.5%) reported at least one type of patient aggression. Additional demographic information is presented in Table 1.

**Table 1:** Demographic characteristics of the participants (*N* = 704).

Variables		n	%
Sex	Female	638	90.6
	Male	66	9.4
Age	≤30	375	53.3
	31–50	316	44.9
	≥51	13	1.8
Working years	≤4	153	21.7
	5–10	316	44.9
	≥11	235	33.4
Marriage	Unmarried	195	27.7
	Married	478	67.9
	Divorced	26	3.7
	Widowed	2	0.3
	Other	3	0.4
Educational attainment	Secondary vocational education	33	4.7
	Junior college	269	38.2
	Bachelor’s degree	398	56.5
	Postgraduate degree	4	0.6
Professional title	No professional title	158	22.4
	Nurse	366	52.0
	Nurse-in-charge	170	24.1
	Associate chief nurse	10	1.4
Department	Internal Medicine	116	16.5
	Surgery	137	19.5
	Gynecology	16	2.3
	Pediatrics	19	2.7
	Psychiatry	331	47.0
	Emergency Department	6	0.9
	Intensive Care Unit	13	1.8
	Operating Room	10	1.4
	Hemodialysis Unit	10	1.4
	Outpatient Department	14	2.0
	Central Sterile Supply Department	9	1.3
	Others	23	3.3
Hospital grade	Secondary grade A hospital	90	12.8
	Tertiary grade B hospital	16	2.3
	Tertiary grade A hospital	598	84.9
Hospital specialty	Psychiatric specialty hospital	331	47.0
	Non-psychiatric hospital	373	53.0
Patient aggression experience	None	130	18.5
	Verbal aggression	268	38.1
	Minor physical aggression	163	23.2
	Moderate physical aggression	140	19.9
	Severe physical aggression	3	0.4

Percentages may not total 100% due to rounding.

Mean scores and standard deviations of the WLEIS dimensions and DASS-21 items are shown in Table 2. A Pearson correlation heatmap showed generally negative correlations between the WLEIS dimension scores and the depression, anxiety, and stress subscale scores (Supplementary Figure S2). Pearson correlations and regularized partial correlations among the 25 network nodes are presented in Supplementary Tables S1, S2, respectively.

**Table 2:** Abbreviations, mean scores, standard deviations, expected influence, and predictability of WLEIS dimensions and DASS-21 items (*N* = 704).

Items	Mean	SD	EI	Predictability
SEA	21.81	3.31	0.98	0.68
OEA	20.57	3.58	0.70	0.52
UOE	21.09	3.58	0.77	0.68
ROE	19.90	4.29	0.36	0.56
DASS_01	0.92	0.69	0.72	0.36
DASS_02	1.17	0.87	0.59	0.30
DASS_03	0.89	0.82	1.03	0.54
DASS_04	0.61	0.76	1.14	0.57
DASS_05	0.74	0.72	0.71	0.37
DASS_06	0.73	0.68	0.98	0.47
DASS_07	0.48	0.69	0.94	0.46
DASS_08	1.33	0.96	0.95	0.46
DASS_09	1.39	0.88	0.53	0.38
DASS_10	0.99	0.86	0.81	0.55
DASS_11	0.86	0.83	1.20	0.68
DASS_12	1.01	0.85	1.01	0.61
DASS_13	0.80	0.80	1.18	0.69
DASS_14	1.09	0.81	0.71	0.32
DASS_15	0.62	0.76	1.16	0.63
DASS_16	0.73	0.73	0.79	0.52
DASS_17	0.19	0.45	0.92	0.40
DASS_18	0.91	0.83	0.64	0.45
DASS_19	0.59	0.79	0.79	0.52
DASS_20	0.56	0.76	1.12	0.57
DASS_21	0.31	0.60	0.96	0.53

Predictability was expressed as R^2^, indicating the proportion of variance in each node explained by its neighboring nodes. WLEIS, Wong and Law Emotional Intelligence Scale; DASS-21, Depression Anxiety Stress Scales-21; SEA, self-emotion appraisal; OEA, others’ emotion appraisal; UOE, use of emotion; ROE, regulation of emotion; EI, expected influence; SD, standard deviation.

### 3.2 Network structure

Figure 1 presents the estimated network comprising the four emotional intelligence dimensions and 21 DASS-21 symptom items among clinical nurses. The network comprised 25 nodes. Of the 300 possible edges, 151 (50.3%) were retained as nonzero after regularization, including 126 positive edges and 25 negative edges. Within the DASS-21 symptom community, the three largest estimated edge weights were DASS_17–DASS_21 (0.350), DASS_19–DASS_20 (0.336), and DASS_05–DASS_06 (0.315). Among the four emotional intelligence dimensions, the largest estimated edge weight was observed between SEA and UOE (0.487), followed by SEA–OEA (0.304) and UOE–ROE (0.236). For the cross-community edges linking emotional intelligence and DASS-21 symptoms, the three largest estimated absolute edge weights were ROE–DASS_18 (−0.186), UOE–DASS_16 (−0.085), and ROE–DASS_09 (−0.068) (Supplementary Table S4). Bootstrapped edge-weight difference tests are presented in Supplementary Figure S3.

**Figure 1:**
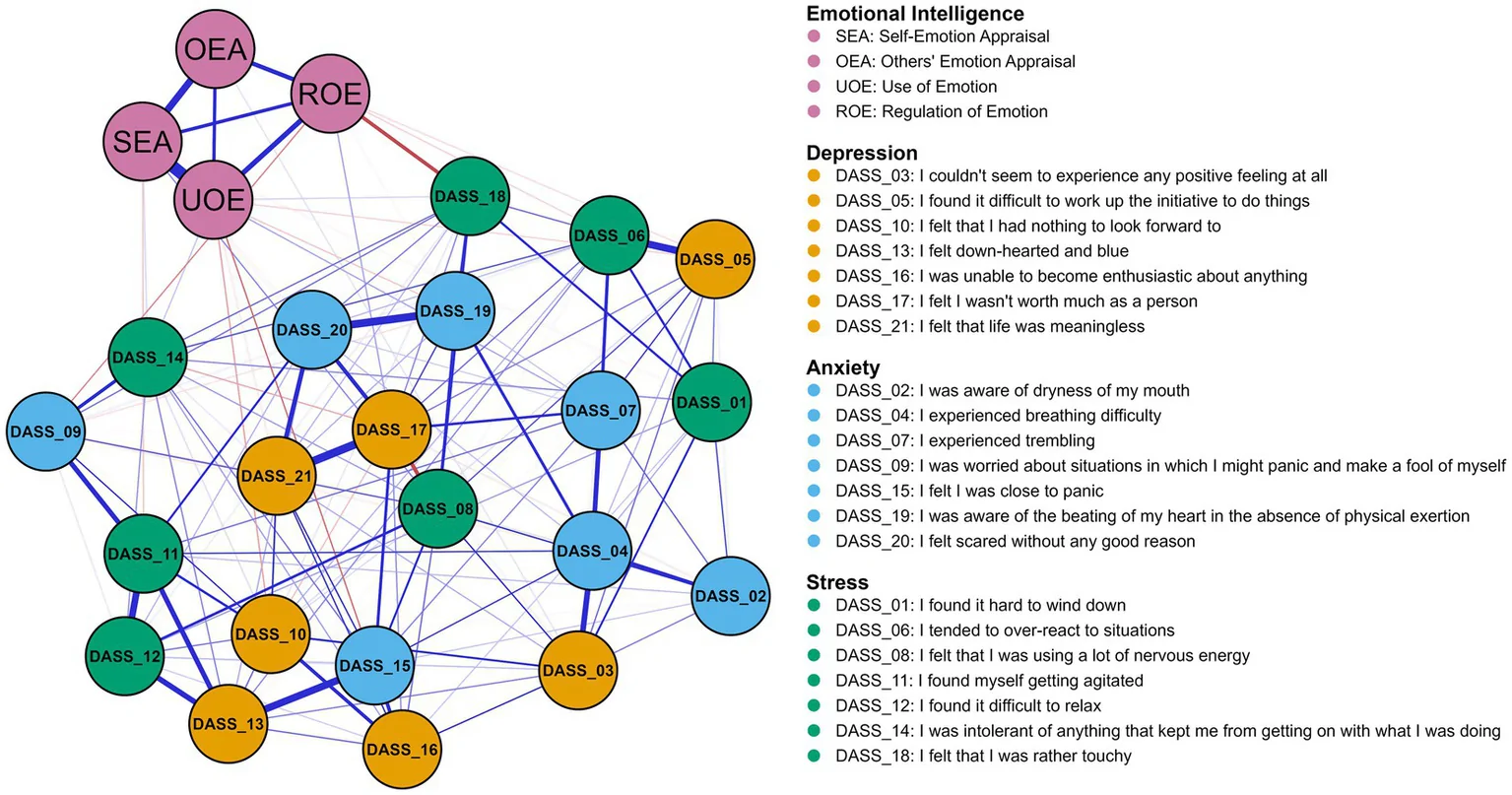
Network structure of emotional intelligence dimensions and depression, anxiety, and stress symptoms. Nodes represent emotional intelligence dimensions and symptoms of depression, anxiety, and stress. Blue edges indicate positive associations, and red edges indicate negative associations. Edge thickness reflects the absolute magnitude of the regularized edge weight.

### 3.3 Node centrality, predictability, and bridge centrality

Figure 2 summarizes the centrality indices of all nodes in the network (Supplementary Table S3). The highest strength value was observed for DASS_17 (1.558), followed by DASS_08 (1.347) and DASS_11 (1.273). For expected influence (EI), DASS_11 had the highest value (1.203), followed by DASS_13 (1.184) and DASS_15 (1.163). Bootstrapped difference tests for expected influence are shown in Supplementary Figure S4. Predictability values ranged from 0.30 to 0.69 across nodes. DASS_13 showed the highest predictability (0.69), followed by UOE, DASS_11, and SEA (all 0.68), whereas DASS_02 showed the lowest value (0.30).

**Figure 2:**
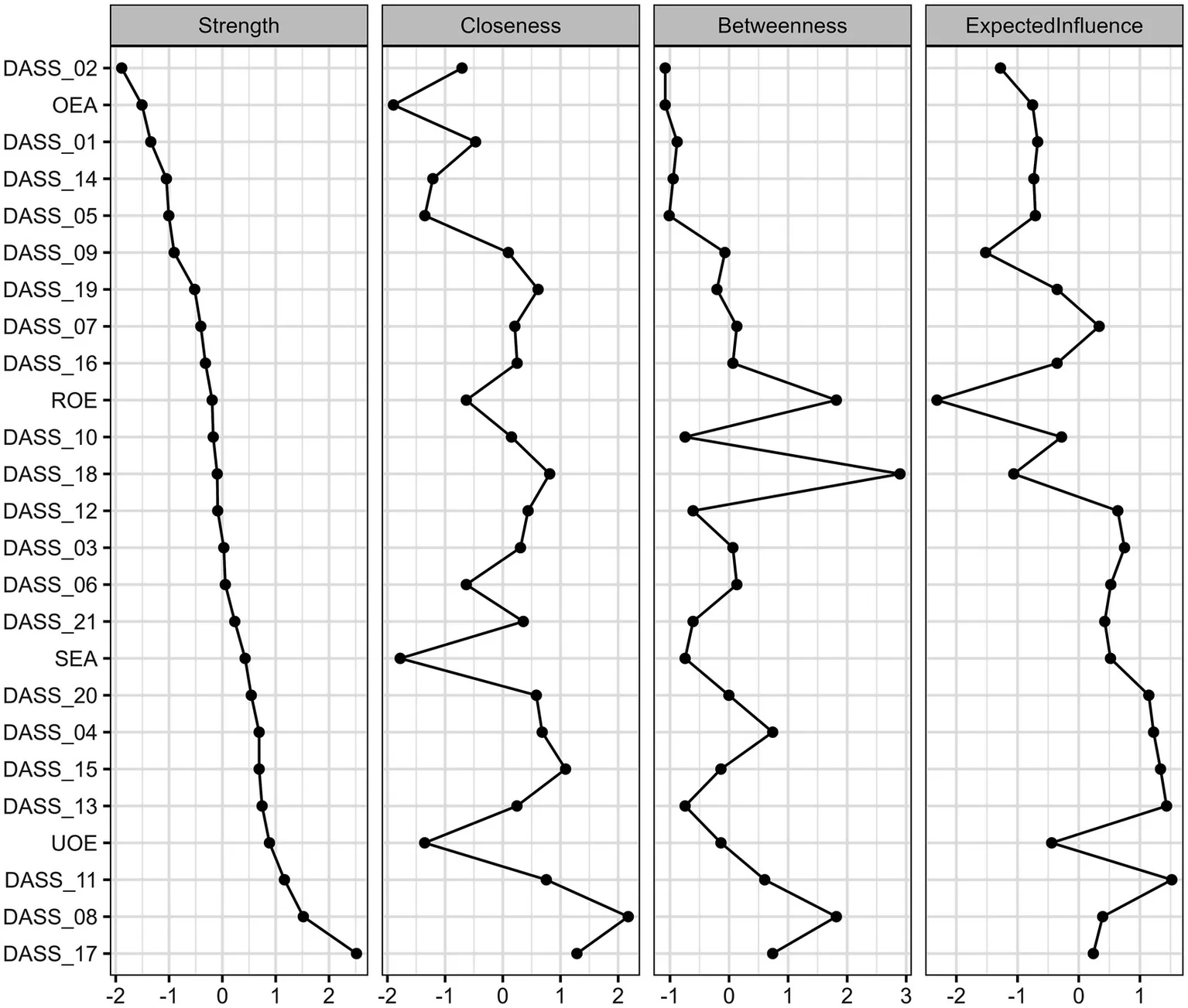
Centrality indices of the emotional intelligence and symptom network. The plot presents strength, closeness, betweenness, and expected influence.

Figure 3 presents the bridge expected influence (BEI) estimates for all nodes (Supplementary Table S3). Among the four WLEIS dimension nodes, ROE had the largest absolute BEI (BEI = −0.268), followed by UOE (BEI = −0.125). Among the DASS-21 symptom nodes, DASS_18 had the largest absolute BEI (BEI = −0.163). Bootstrapped difference tests for BEI are presented in Supplementary Figure S5.

**Figure 3:**
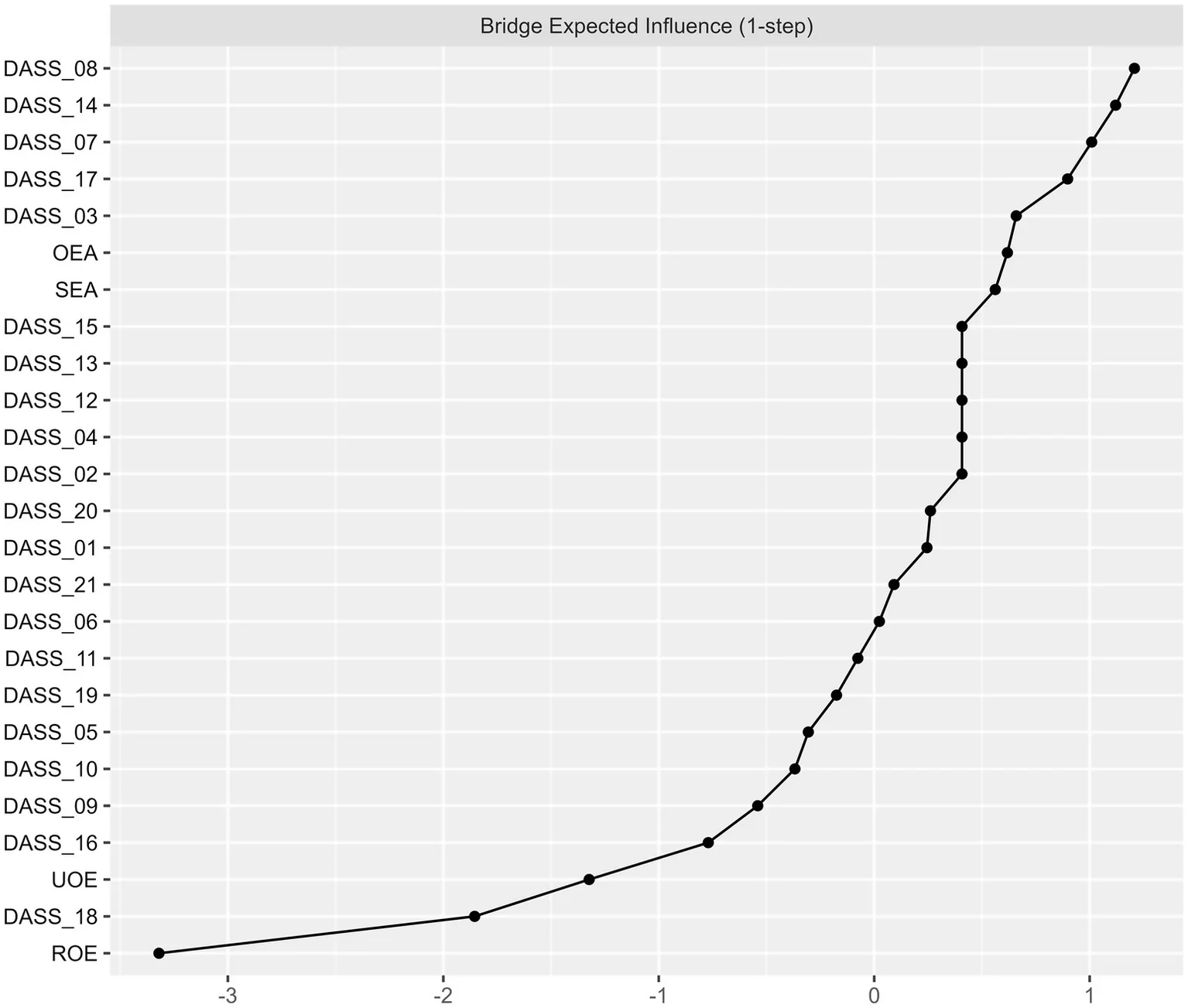
Bridge expected influence in the emotional intelligence and symptom network. Bridge expected influence indicates the extent to which a node connects emotional intelligence dimensions with depression, anxiety, and stress symptoms.

### 3.4 Stability and precision of the network

Figure 4 shows that most edge-weight estimates were accompanied by relatively narrow 95% confidence intervals, indicating adequate precision. The stability analysis showed that the CS-coefficients for strength, EI, and BEI were 0.52, 0.75, and 0.67, respectively. As all three coefficients exceeded 0.50, these indices were considered to have good stability (Figure 5).

**Figure 4:**
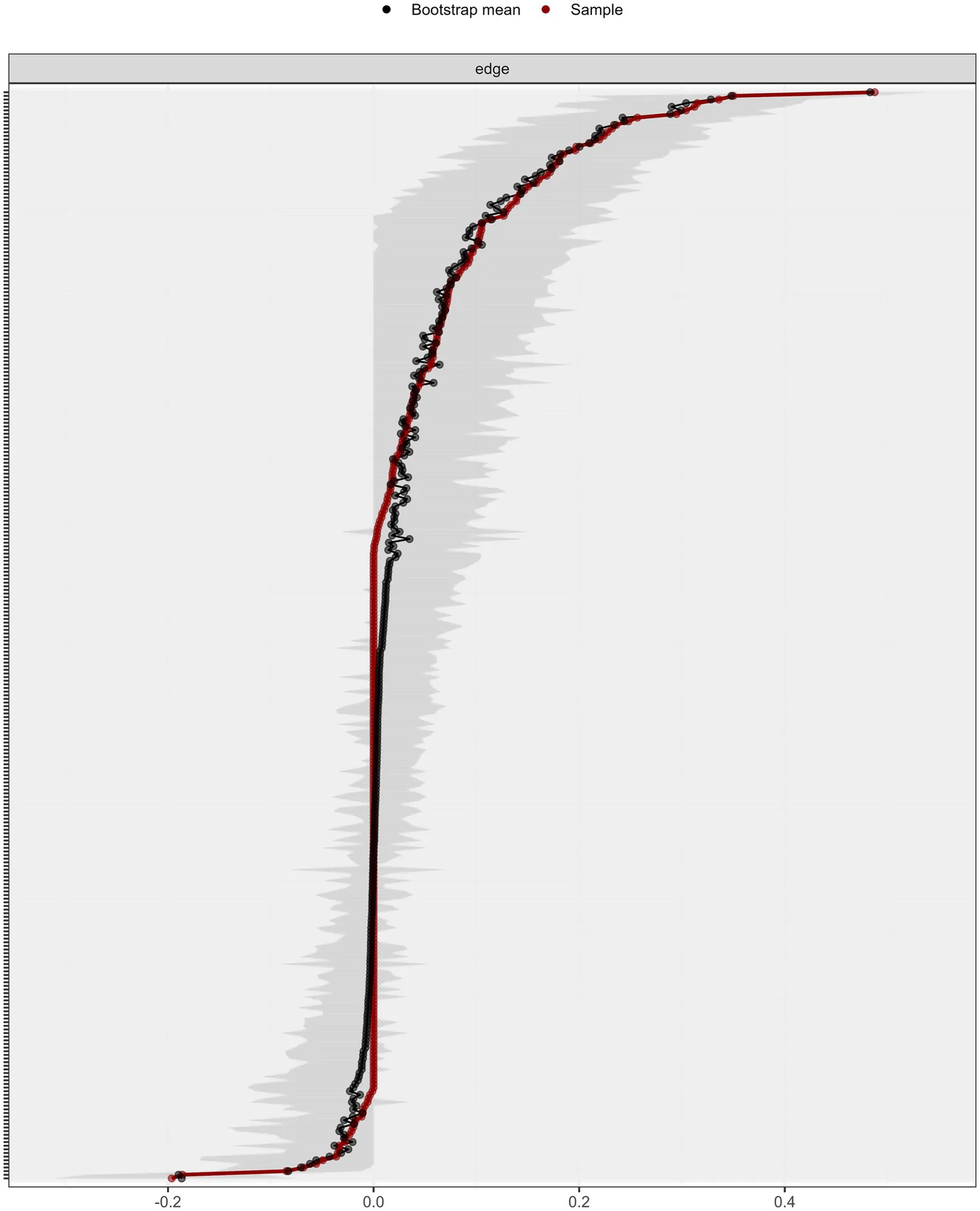
Accuracy of edge weights in the emotional intelligence and symptom network. Gray areas represent bootstrap 95% confidence intervals. Narrower intervals indicate greater precision of the estimated edges.

**Figure 5:**
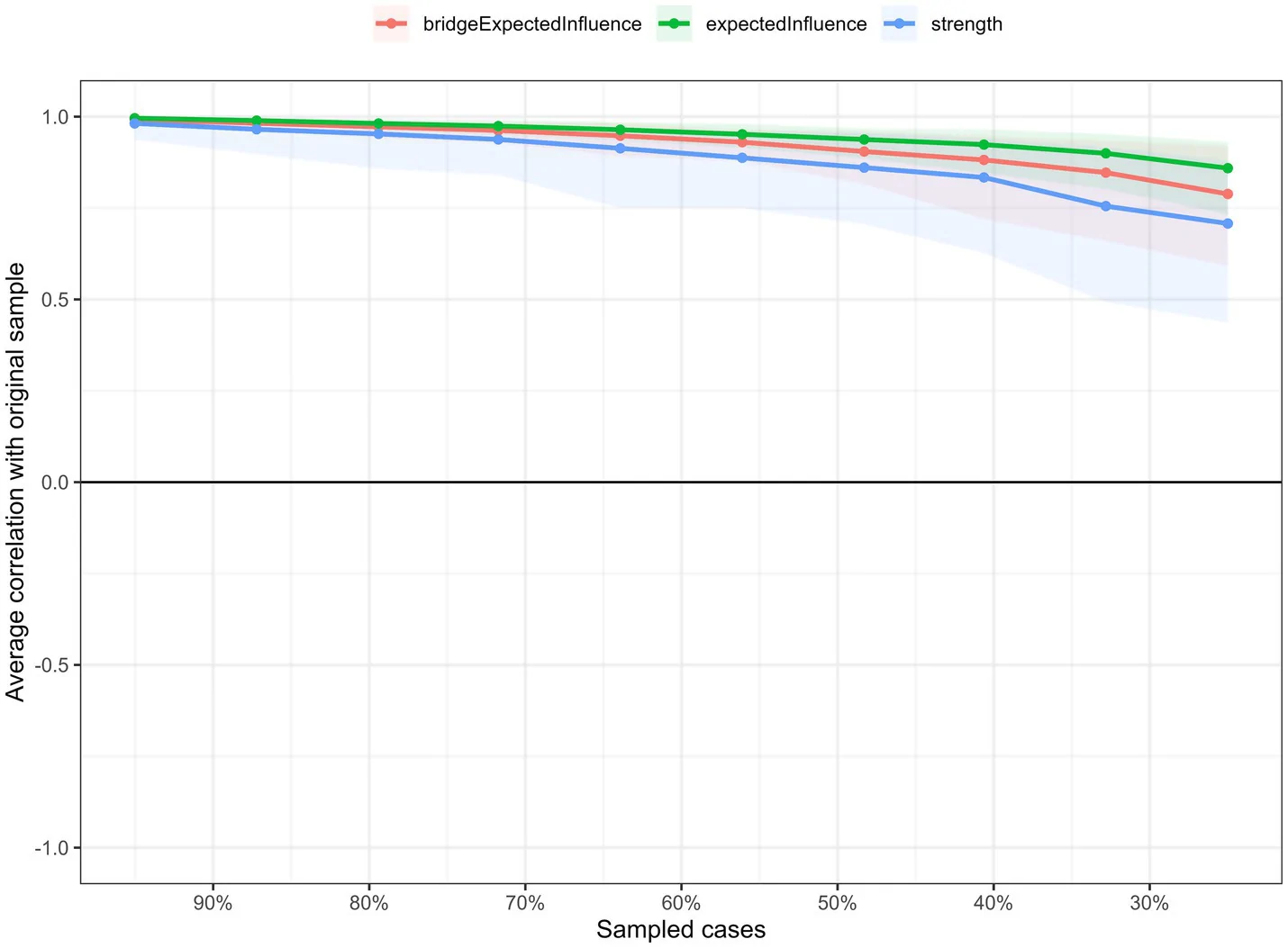
Case-dropping bootstrap results for centrality indices and bridge expected influence. The plot shows the stability of strength, expected influence, and bridge expected influence.

### 3.5 Network comparison

To assess whether patient aggression experience was associated with differences in the network linking emotional intelligence and negative affective symptoms, the networks of nurses with and without patient aggression experience were compared using the Network Comparison Test (NCT). The NCT did not detect statistically significant between-group differences in overall network structure (*M* = 0.250, *p* = 0.489) or global strength (*S* = 0.194, *p* = 0.869). No individual edge difference remained statistically significant after Benjamini–Hochberg FDR correction. The estimated networks for the two groups are shown for descriptive purposes (Supplementary Figure S8), while between-group differences were evaluated statistically using the NCT.

The *post hoc* exploratory analysis further compared psychiatric nurses (*n* = 331) with non-psychiatric nurses (*n* = 373). The Network Comparison Test did not detect statistically significant between-group differences in overall network structure (*M* = 0.244, *p* = 0.472) or global strength (*S* = 0.535, *p* = 0.478). After Benjamini–Hochberg correction, no between-group difference in any individual edge reached statistical significance.

### 3.6 Sensitivity analyses

The main network findings remained stable after separate adjustment for age and years of work experience. In the network adjusted for age and the network adjusted for years of work experience, ROE–DASS_18, UOE–DASS_16, and ROE–DASS_09 remained the three cross-community edges with the largest absolute weights. DASS_11 retained the highest expected influence, and ROE retained the largest absolute bridge expected influence (BEI). In both networks, the CS coefficients for strength, expected influence, and bridge expected influence (BEI) were 0.75, 0.75, and 0.67, respectively. Detailed results are presented in Supplementary Tables S5, S6 and Supplementary Figures S6, S7.

## 4 Discussion

This study examined symptom-level conditional associations between emotional intelligence dimensions and symptoms of depression, anxiety, and stress among clinical nurses. Three main findings emerged. First, cross-community associations between emotional intelligence dimensions and negative affective symptoms were concentrated in a small number of specific nodes. ROE–DASS_18 had the largest absolute cross-community edge weight. Second, DASS_11 had the highest expected influence among the DASS-21 symptom nodes. Among the four WLEIS dimension nodes, ROE had the largest absolute bridge expected influence (BEI). Its negative BEI indicates that the sum of its cross-community edge weights was negative within the current network and community specification. Third, the Network Comparison Test did not detect statistically significant differences in overall network structure or global strength between nurses with and without patient aggression experience. However, this finding should be interpreted cautiously because the subgroup sizes were markedly imbalanced. Overall, by extending previous work based on total-score or dimension-level analyses, this study identified conditional associations between emotional intelligence dimensions and specific negative affective symptoms. These findings identify candidate variables that warrant further examination in longitudinal or intervention studies.

### 4.1 Main findings

Consistent with previous studies in nurse samples, emotional intelligence was generally negatively associated with depression, anxiety, and stress symptoms (Abualruz et al., 2024; Sun et al., 2021). However, the present network showed that the corresponding symptom-level conditional associations were not evenly distributed across all nodes. Rather, they were concentrated in a small number of specific symptoms and emotional intelligence dimensions. This pattern suggests that network analysis can provide more specific symptom-level information than total-score approaches when examining the relationship between emotional intelligence and negative affective symptoms in clinical nurses (Briganti et al., 2024).

Among the DASS-21 symptom nodes, DASS_11 (“I found myself getting agitated”) had the highest expected influence and the third-highest strength. This indicates that DASS_11 had relatively strong overall conditional connectivity in the current network. DASS_11 is part of the stress subscale and primarily reflects agitation. Its high connectivity may be related to the sustained cognitive and emotional demands of clinical nursing. Clinical nurses must closely monitor changes in patients’ conditions, respond promptly to unexpected events, and manage potential risks during care. Previous studies have linked occupational stress to poorer mental health among clinical nurses. Moderate levels of occupational stress have also been reported among nurses in China (Jin F. et al., 2025; Zhong et al., 2025). This evidence provides relevant occupational context for understanding the high connectivity of DASS_11 in the present network.

A previous network study of nurses found that the stress dimension had the highest expected influence and strength (Tao et al., 2024), which is broadly consistent with the prominent position of DASS_11 in the present network. The relatively high connectivity of DASS_11 may also be related to the emotional labor involved in clinical nursing. When communicating with patients and their families, nurses often need to regulate their emotional expression while continuing to perform professionally in high-pressure clinical settings. Previous research has shown that emotional labor is associated with psychological burden and work-related outcomes among nurses (Feng et al., 2024). In addition, DASS_13 (“I felt down-hearted and blue”) and DASS_15 (“I felt I was close to panic”) showed relatively high expected influence values among the DASS-21 symptom nodes, and each showed conditional associations with multiple symptoms across the depression, anxiety, and stress domains. Previous network studies of nurses similarly found that low mood and anxiety-related symptoms, including fear, worry, and difficulty relaxing, may occupy relatively central positions in symptom networks (Peng et al., 2022; Xue et al., 2026).

Following the identification of central symptoms, analyses of bridge centrality and cross-community edges provided further information on the conditional associations between emotional intelligence and negative affective symptoms. Among these associations, the negative edge between ROE (“Regulation of emotion”) and DASS_18 (“I felt that I was rather touchy”) had the largest absolute weight of all cross-community edges. DASS_18 primarily reflects irritability and heightened emotional reactivity. After controlling for all other nodes in the network, higher ROE scores were conditionally associated with lower DASS_18 scores. Research indicates that emotion regulation involves modifying the intensity, duration, and expression of emotional responses (McRae and Gross, 2020). In clinical nursing, patients’ suffering, concerns expressed by family members, communication-related conflicts, and unexpected events may evoke negative emotions. In these high-pressure interpersonal situations, difficulties in emotion regulation may occur alongside greater irritability and stronger emotional responses among nurses. Previous research has also shown that irritability is closely associated with difficulties in emotion regulation, as well as anxiety and depression (Leibenluft et al., 2024). A meta-analysis further found that anger was positively associated with maladaptive emotion regulation strategies and negatively associated with adaptive emotion regulation strategies (Pop et al., 2025). Taken together, these studies indicate a close association between emotion regulation ability and irritability, which is consistent with the negative conditional association observed between ROE and DASS_18 in the present study.

In addition, two other negative cross-community edges with relatively large absolute weights were observed: UOE–DASS_16 (“I was unable to become enthusiastic about anything”) and ROE–DASS_09 (“I was worried about situations in which I might panic and make a fool of myself”). UOE refers to the ability to use emotions to facilitate action and goal pursuit, whereas DASS_16 reflects reduced enthusiasm (Wong and Law, 2002). Positive emotions are associated with active engagement, while anhedonia is often accompanied by reduced motivation and enthusiasm. This is broadly consistent with the negative conditional association observed between UOE and DASS_16 in the present network (Fredrickson, 2001; Wu et al., 2025). The ROE–DASS_09 edge indicated that higher ROE scores were conditionally associated with lower levels of worry about situations in which one might panic and make a fool of oneself. DASS_09 primarily reflects situational anxiety involving anticipatory worry and social embarrassment. Previous studies have linked difficulties in emotion regulation to social anxiety and anxiety symptom levels (Dryman and Heimberg, 2018; Reichler et al., 2025). Given their relatively small magnitudes, these edges should be viewed as supplementary and interpreted cautiously.

Among the four WLEIS dimensions, ROE had the largest absolute BEI and appeared in two of the three cross-community edges with the largest absolute weights. Bridge expected influence is used to identify nodes with prominent connections between different symptom or psychological construct communities (Jones et al., 2021). Thus, within the current network and community specification, ROE showed more prominent overall cross-community conditional associations with the DASS-21 symptom community than the other emotional intelligence dimensions. Clinical nurses often encounter situations that may elicit negative emotions during patient care, communication, and the management of unexpected clinical events. A recent systematic review indicated that reducing negative emotions is an important goal of emotion regulation (Martínez-Priego et al., 2024). The prominent bridge pattern of ROE may be related to the need for nurses to continually regulate their emotional responses in clinical practice. Nursing studies have also reported associations of emotional intelligence and emotion-management self-efficacy with negative psychological states and job burnout among nurses (Szczygiel and Mikolajczak, 2018; Yu et al., 2023). In addition, different emotion regulation profiles have been associated with levels of depressive and anxiety symptoms (Reichler et al., 2025). Taken together, these findings highlight ROE as a candidate variable for further investigation, although its associations with negative affective symptoms require further examination in longitudinal or intervention studies.

In addition, the exploratory network comparisons revealed no statistically significant differences in overall network structure or global strength between nurses with and without patient aggression experience, or between psychiatric and non-psychiatric nurses. These findings should not be interpreted as evidence that the corresponding networks were equivalent. The dichotomization of patient aggression experience may have obscured differences associated with the type and severity of aggression, while the marked imbalance in subgroup sizes may have limited the detection of smaller network differences. Regarding nursing work settings, previous studies have suggested that psychiatric nursing may be associated with differences in certain emotional intelligence characteristics and emotion regulation-related psychological processes (Ishii and Horikawa, 2019; Jin T. et al., 2025). However, such differences may be more apparent in specific psychological characteristics or particular variable-level associations and may not necessarily translate into detectable changes in the overall network structure linking emotional intelligence dimensions with specific negative affective symptoms.

Notably, the present data were collected during the COVID-19 pandemic, when pandemic-related workload and psychological stress may have influenced some negative affective symptoms and their network characteristics. A network study of Chinese nurses conducted during the pandemic similarly found that symptoms such as restlessness and difficulty relaxing showed relatively high network importance (Peng et al., 2022), which is broadly consistent with the prominent network position of DASS_11 in the present study. A subsequent longitudinal network study further showed that although the centrality of some nodes and overall network connectivity varied across periods before and during the pandemic, no statistically significant difference was observed in the overall network structure (Blackmore et al., 2026), suggesting that the pandemic context may have influenced some local network characteristics without necessarily altering the overall organization of symptom relationships. Meanwhile, the relationship between nurses’ emotional abilities and negative affective symptoms has remained an important topic in nursing mental health research in recent years. Therefore, although the data collection context should be considered when interpreting specific network characteristics in the present study, our findings may still provide useful evidence for understanding the pattern of associations between emotional intelligence and negative affective symptoms among nurses.

### 4.2 Clinical implications

The findings may provide symptom-level guidance for mental health assessment among nurses. DASS_11 had the highest expected influence, suggesting that agitation may warrant attention during assessment. DASS_13 and DASS_15 also showed relatively high expected influence, indicating that experiences of low mood and feeling close to panic may also be considered. Compared with total or subscale scores, network analysis can provide complementary information about specific symptoms and their connections with other nodes (Briganti et al., 2024). In the present study, this symptom-level information helped identify symptoms and emotional intelligence dimensions that may warrant attention in assessment and future research. Timely assessment and follow-up may be considered for nurses who report these symptoms following patient aggression or adverse clinical events, as such experiences may adversely affect the mental health of healthcare workers (Ong et al., 2025; Yosep et al., 2023). Given its prominent bridge position, ROE may be considered a candidate dimension for further examination in future research on psychological support. Existing evidence suggests that emotional intelligence training may help reduce stress and improve communication skills among nurses and nursing students (Kawashima et al., 2025), while broader research indicates that emotion regulation interventions may improve mental health-related outcomes (Saccaro et al., 2024).

## 5 Limitations

This study has several limitations. First, the cross-sectional design precludes determination of the direction or causality of the conditional associations between nodes, and centrality and bridge indices cannot be used directly to identify intervention targets. Second, the emotional intelligence dimension nodes and the depression, anxiety, and stress symptom-item nodes differed in their levels of aggregation, community size, and measurement properties, which may have influenced the network topology and the estimation of related indices. In addition, the sensitivity analyses separately considered only age and years of work experience and therefore could not rule out the influence of other factors. Third, the use of convenience sampling and self-report measures may have introduced selection bias and common method bias. Because information that could reliably identify individual hospitals was unavailable, hospital-level clustering and the potential influence of shared organizational environments could not be further evaluated. Moreover, restricting submissions by IP address could not completely exclude duplicate participation and may also have prevented some eligible nurses sharing the same network from responding. Fourth, the sample was drawn mainly from southwestern China and included relatively high proportions of women and psychiatric nurses, which may limit the generalizability of the findings. In addition, most participants worked in tertiary grade A hospitals; caution is warranted when generalizing the findings to nurses working in lower-level hospitals. Because nurses with a self-reported history of psychiatric or psychological disorders were excluded to reduce heterogeneity related to pre-existing mental health conditions, the findings may not be directly generalizable to nurses with such histories, including those who may have elevated symptom levels. Future studies could investigate whether these network characteristics are also observed among nurses with such histories. Fifth, dichotomizing patient aggression experience may have obscured differences related to the type and severity of aggression, while the imbalance in subgroup sizes may have limited the ability to detect smaller between-group network differences. Sixth, the data were collected during the COVID-19 pandemic, when pandemic-related workload and psychological stress may have influenced some negative affective symptoms and their network characteristics. Future studies are therefore needed to examine these findings across different time periods and in more diverse nursing populations.

## 6 Conclusion

Using network analysis, this study characterized symptom-level conditional associations between emotional intelligence dimensions and symptoms of depression, anxiety, and stress among clinical nurses, providing a more fine-grained perspective on their relationships. Agitation, as reflected by DASS_11, and emotion regulation, as represented by ROE, showed relatively prominent connectivity patterns in the current network and may be considered candidate variables for further investigation in research on nurses’ mental health assessment and psychological support. Longitudinal and intervention studies are needed to further examine these associations and their potential clinical relevance.

## Data Availability

The datasets analyzed during the current study are not publicly available due to privacy and ethical restrictions but are available from the corresponding author on reasonable request.
