# Supplementary material for: Emotional intelligence and depression, anxiety, and stress symptoms among clinical nurses: a cross-sectional network analysis
Source: Front Psychol. 2026 Sep 30;17:1894001. doi: 10.3389/fpsyg.2026.1894001 (PMC13661961; doi:10.3389/fpsyg.2026.1894001)
Supplement: Supplementary file 1 [file Supplementary_file_1.docx]

Supplementary Material

| Supplementary Table S1. Pearson correlation matrix of emotional intelligence dimensions and DASS-21 symptom nodes | | | | | | | | | | | | | | | | | | | | | | | | | |
| --- | --- | --- | --- | --- | --- | --- | --- | --- | --- | --- | --- | --- | --- | --- | --- | --- | --- | --- | --- | --- | --- | --- | --- | --- | --- |
|  | SEA | OEA | UOE | ROE | DASS_01 | DASS_02 | DASS_03 | DASS_04 | DASS_05 | DASS_06 | DASS_07 | DASS_08 | DASS_09 | DASS_10 | DASS_11 | DASS_12 | DASS_13 | DASS_14 | DASS_15 | DASS_16 | DASS_17 | DASS_18 | DASS_19 | DASS_20 | DASS_21 |
| SEA |  |  |  |  |  |  |  |  |  |  |  |  |  |  |  |  |  |  |  |  |  |  |  |  |  |
| OEA | .682** |  |  |  |  |  |  |  |  |  |  |  |  |  |  |  |  |  |  |  |  |  |  |  |  |
| UOE | .784** | .646** |  |  |  |  |  |  |  |  |  |  |  |  |  |  |  |  |  |  |  |  |  |  |  |
| ROE | .650** | .601** | .666** |  |  |  |  |  |  |  |  |  |  |  |  |  |  |  |  |  |  |  |  |  |  |
| DASS_01 | -.230** | -.174** | -.220** | -.243** |  |  |  |  |  |  |  |  |  |  |  |  |  |  |  |  |  |  |  |  |  |
| DASS_02 | -.160** | -.166** | -.175** | -.204** | .378** |  |  |  |  |  |  |  |  |  |  |  |  |  |  |  |  |  |  |  |  |
| DASS_03 | -.198** | -.148** | -.251** | -.269** | .478** | .451** |  |  |  |  |  |  |  |  |  |  |  |  |  |  |  |  |  |  |  |
| DASS_04 | -.232** | -.185** | -.283** | -.286** | .432** | .486** | .616** |  |  |  |  |  |  |  |  |  |  |  |  |  |  |  |  |  |  |
| DASS_05 | -.228** | -.192** | -.278** | -.289** | .394** | .347** | .459** | .441** |  |  |  |  |  |  |  |  |  |  |  |  |  |  |  |  |  |
| DASS_06 | -.233** | -.212** | -.275** | -.316** | .459** | .335** | .484** | .476** | .561** |  |  |  |  |  |  |  |  |  |  |  |  |  |  |  |  |
| DASS_07 | -.155** | -.126** | -.173** | -.194** | .359** | .391** | .465** | .584** | .431** | .500** |  |  |  |  |  |  |  |  |  |  |  |  |  |  |  |
| DASS_08 | -.143** | -.167** | -.225** | -.230** | .388** | .423** | .519** | .473** | .416** | .427** | .376** |  |  |  |  |  |  |  |  |  |  |  |  |  |  |
| DASS_09 | -.207** | -.191** | -.262** | -.322** | .303** | .311** | .379** | .330** | .287** | .379** | .303** | .434** |  |  |  |  |  |  |  |  |  |  |  |  |  |
| DASS_10 | -.266** | -.205** | -.366** | -.322** | .398** | .340** | .571** | .454** | .426** | .475** | .422** | .490** | .470** |  |  |  |  |  |  |  |  |  |  |  |  |
| DASS_11 | -.317** | -.223** | -.336** | -.343** | .435** | .444** | .582** | .602** | .423** | .491** | .518** | .513** | .538** | .641** |  |  |  |  |  |  |  |  |  |  |  |
| DASS_12 | -.251** | -.206** | -.295** | -.287** | .452** | .427** | .576** | .523** | .402** | .473** | .425** | .564** | .481** | .604** | .733** |  |  |  |  |  |  |  |  |  |  |
| DASS_13 | -.281** | -.209** | -.327** | -.328** | .437** | .424** | .600** | .577** | .462** | .534** | .515** | .517** | .449** | .624** | .732** | .718** |  |  |  |  |  |  |  |  |  |
| DASS_14 | -.111** | -.095* | -.140** | -.198** | .351** | .285** | .396** | .342** | .312** | .394** | .305** | .412** | .428** | .408** | .428** | .457** | .461** |  |  |  |  |  |  |  |  |
| DASS_15 | -.239** | -.187** | -.277** | -.262** | .430** | .422** | .561** | .608** | .426** | .491** | .531** | .517** | .357** | .560** | .641** | .611** | .718** | .430** |  |  |  |  |  |  |  |
| DASS_16 | -.290** | -.228** | -.372** | -.288** | .415** | .342** | .561** | .501** | .404** | .454** | .424** | .486** | .360** | .596** | .575** | .569** | .619** | .405** | .625** |  |  |  |  |  |  |
| DASS_17 | -.215** | -.165** | -.226** | -.177** | .283** | .222** | .348** | .398** | .324** | .381** | .439** | .217** | .215** | .319** | .400** | .359** | .426** | .210** | .477** | .398** |  |  |  |  |  |
| DASS_18 | -.277** | -.228** | -.275** | -.421** | .438** | .358** | .443** | .450** | .379** | .465** | .394** | .490** | .405** | .426** | .507** | .496** | .509** | .392** | .488** | .423** | .348** |  |  |  |  |
| DASS_19 | -.254** | -.204** | -.312** | -.310** | .344** | .338** | .480** | .566** | .359** | .445** | .474** | .438** | .292** | .426** | .524** | .481** | .501** | .302** | .521** | .463** | .448** | .474** |  |  |  |
| DASS_20 | -.279** | -.222** | -.305** | -.323** | .377** | .377** | .517** | .579** | .414** | .488** | .519** | .399** | .381** | .479** | .619** | .559** | .606** | .335** | .587** | .468** | .545** | .491** | .658** |  |  |
| DASS_21 | -.219** | -.207** | -.289** | -.267** | .321** | .270** | .453** | .447** | .368** | .400** | .434** | .332** | .263** | .482** | .477** | .441** | .518** | .320** | .562** | .496** | .601** | .412** | .501** | .612** |  |
| ***p* < .01; **p* < .05.  *Note.* SEA: Self-Emotion Appraisal; OEA: Others' Emotion Appraisal; UOE: Use of Emotion; ROE: Regulation of Emotion; DASS_01: Difficulty winding down; DASS_02: Dry mouth; DASS_03: Lack of positive feelings; DASS_04: Breathing difficulty; DASS_05: Lack of initiative; DASS_06: Overreactivity; DASS_07: Trembling; DASS_08: Nervous energy; DASS_09: Panic-related worry; DASS_10: Nothing to look forward to; DASS_11: Agitation; DASS_12: Difficulty relaxing; DASS_13: Low mood; DASS_14: Intolerance of interruptions; DASS_15: Feeling close to panic; DASS_16: Lack of enthusiasm; DASS_17: Low self-worth; DASS_18: Irritability; DASS_19: Awareness of heartbeat; DASS_20: Unexplained fear; DASS_21: Meaninglessness. | | | | | | | | | | | | | | | | | | | | | | | | | |

| Supplementary Table S2. Regularized partial correlation matrix of emotional intelligence dimensions and DASS-21 symptom nodes | | | | | | | | | | | | | | | | | | | | | | | | | |
| --- | --- | --- | --- | --- | --- | --- | --- | --- | --- | --- | --- | --- | --- | --- | --- | --- | --- | --- | --- | --- | --- | --- | --- | --- | --- |
|  | SEA | OEA | UOE | ROE | D01 | D02 | D03 | D04 | D05 | D06 | D07 | D08 | D09 | D10 | D11 | D12 | D13 | D14 | D15 | D16 | D17 | D18 | D19 | D20 | D21 |
| SEA |  |  |  |  |  |  |  |  |  |  |  |  |  |  |  |  |  |  |  |  |  |  |  |  |  |
| OEA | 0.304 |  |  |  |  |  |  |  |  |  |  |  |  |  |  |  |  |  |  |  |  |  |  |  |  |
| UOE | 0.487 | 0.172 |  |  |  |  |  |  |  |  |  |  |  |  |  |  |  |  |  |  |  |  |  |  |  |
| ROE | 0.180 | 0.211 | 0.236 |  |  |  |  |  |  |  |  |  |  |  |  |  |  |  |  |  |  |  |  |  |  |
| D01 | -0.012 | 0.000 | 0.000 | 0.000 |  |  |  |  |  |  |  |  |  |  |  |  |  |  |  |  |  |  |  |  |  |
| D02 | 0.000 | 0.000 | 0.000 | 0.000 | 0.089 |  |  |  |  |  |  |  |  |  |  |  |  |  |  |  |  |  |  |  |  |
| D03 | 0.000 | 0.018 | 0.000 | 0.000 | 0.127 | 0.063 |  |  |  |  |  |  |  |  |  |  |  |  |  |  |  |  |  |  |  |
| D04 | 0.000 | 0.000 | 0.000 | 0.000 | 0.039 | 0.196 | 0.257 |  |  |  |  |  |  |  |  |  |  |  |  |  |  |  |  |  |  |
| D05 | 0.000 | 0.000 | -0.028 | -0.024 | 0.065 | 0.013 | 0.073 | 0.035 |  |  |  |  |  |  |  |  |  |  |  |  |  |  |  |  |  |
| D06 | 0.000 | 0.000 | 0.000 | -0.028 | 0.139 | 0.000 | 0.011 | 0.000 | 0.315 |  |  |  |  |  |  |  |  |  |  |  |  |  |  |  |  |
| D07 | 0.000 | 0.000 | 0.043 | 0.000 | 0.000 | 0.076 | 0.000 | 0.224 | 0.093 | 0.172 |  |  |  |  |  |  |  |  |  |  |  |  |  |  |  |
| D08 | 0.058 | 0.000 | 0.000 | 0.000 | 0.000 | 0.103 | 0.105 | 0.018 | 0.083 | 0.008 | 0.000 |  |  |  |  |  |  |  |  |  |  |  |  |  |  |
| D09 | 0.000 | 0.000 | 0.000 | -0.068 | 0.000 | 0.000 | 0.000 | -0.017 | 0.000 | 0.013 | 0.000 | 0.081 |  |  |  |  |  |  |  |  |  |  |  |  |  |
| D10 | 0.000 | 0.000 | -0.056 | 0.000 | 0.000 | 0.000 | 0.128 | 0.000 | 0.019 | 0.036 | 0.000 | 0.020 | 0.101 |  |  |  |  |  |  |  |  |  |  |  |  |
| D11 | -0.035 | 0.000 | 0.000 | 0.000 | 0.000 | 0.033 | 0.000 | 0.097 | 0.000 | 0.000 | 0.070 | 0.000 | 0.227 | 0.142 |  |  |  |  |  |  |  |  |  |  |  |
| D12 | 0.000 | 0.000 | 0.000 | 0.000 | 0.047 | 0.006 | 0.032 | 0.000 | 0.000 | 0.000 | 0.000 | 0.150 | 0.017 | 0.066 | 0.295 |  |  |  |  |  |  |  |  |  |  |
| D13 | 0.000 | 0.000 | 0.000 | 0.000 | 0.000 | 0.000 | 0.062 | 0.000 | 0.005 | 0.056 | 0.004 | 0.000 | 0.000 | 0.072 | 0.232 | 0.248 |  |  |  |  |  |  |  |  |  |
| D14 | 0.021 | 0.000 | 0.030 | 0.000 | 0.058 | 0.000 | 0.003 | 0.000 | 0.000 | 0.085 | 0.000 | 0.048 | 0.177 | 0.020 | 0.000 | 0.053 | 0.061 |  |  |  |  |  |  |  |  |
| D15 | 0.000 | 0.000 | 0.000 | 0.000 | 0.000 | 0.029 | 0.000 | 0.096 | 0.000 | 0.000 | 0.059 | 0.131 | -0.005 | 0.000 | 0.008 | 0.018 | 0.312 | 0.068 |  |  |  |  |  |  |  |
| D16 | 0.000 | 0.000 | -0.085 | 0.000 | 0.028 | 0.000 | 0.105 | 0.000 | 0.000 | 0.000 | 0.000 | 0.075 | 0.000 | 0.181 | 0.000 | 0.026 | 0.074 | 0.046 | 0.185 |  |  |  |  |  |  |
| D17 | -0.010 | 0.000 | 0.000 | 0.046 | 0.003 | -0.020 | 0.000 | 0.000 | 0.063 | 0.070 | 0.147 | -0.196 | -0.006 | -0.036 | 0.000 | 0.000 | 0.000 | -0.050 | 0.158 | 0.056 |  |  |  |  |  |
| D18 | 0.000 | 0.000 | 0.023 | -0.186 | 0.139 | 0.001 | 0.000 | 0.000 | 0.000 | 0.072 | 0.000 | 0.168 | 0.067 | 0.000 | 0.000 | 0.014 | 0.017 | 0.071 | 0.000 | 0.000 | 0.058 |  |  |  |  |
| D19 | 0.000 | 0.000 | -0.034 | -0.008 | 0.000 | 0.000 | 0.000 | 0.161 | 0.000 | 0.001 | 0.028 | 0.105 | -0.021 | 0.000 | 0.000 | 0.000 | 0.000 | 0.000 | 0.000 | 0.000 | 0.092 | 0.093 |  |  |  |
| D20 | -0.011 | 0.000 | 0.000 | 0.000 | 0.000 | 0.000 | 0.010 | 0.032 | 0.000 | 0.034 | 0.026 | 0.000 | 0.002 | 0.000 | 0.134 | 0.037 | 0.041 | 0.000 | 0.000 | 0.000 | 0.197 | 0.061 | 0.336 |  |  |
| D21 | 0.000 | -0.003 | -0.019 | 0.000 | 0.000 | -0.001 | 0.040 | 0.000 | 0.000 | 0.000 | 0.000 | 0.000 | -0.033 | 0.114 | 0.000 | 0.000 | 0.000 | 0.016 | 0.104 | 0.102 | 0.350 | 0.036 | 0.039 | 0.220 |  |
| *Note.* Edge weights represent the size and direction of the link between two nodes. SEA: Self-Emotion Appraisal; OEA: Others' Emotion Appraisal; UOE: Use of Emotion; ROE: Regulation of Emotion. D01–D21 denote DASS_01–DASS_21, respectively. DASS_01: Difficulty winding down; DASS_02: Dry mouth; DASS_03: Lack of positive feelings; DASS_04: Breathing difficulty; DASS_05: Lack of initiative; DASS_06: Overreactivity; DASS_07: Trembling; DASS_08: Nervous energy; DASS_09: Panic-related worry; DASS_10: Nothing to look forward to; DASS_11: Agitation; DASS_12: Difficulty relaxing; DASS_13: Low mood; DASS_14: Intolerance of interruptions; DASS_15: Feeling close to panic; DASS_16: Lack of enthusiasm; DASS_17: Low self-worth; DASS_18: Irritability; DASS_19: Awareness of heartbeat; DASS_20: Unexplained fear; DASS_21: Meaninglessness. | | | | | | | | | | | | | | | | | | | | | | | | | |

| Node | Strength | Expected Influence | Bridge Strength | Bridge Expected Influence |
| --- | --- | --- | --- | --- |
| ROE | 0.987 | 0.359 | 0.360 | -0.268 |
| DASS_18 | 1.007 | 0.635 | 0.209 | -0.163 |
| UOE | 1.214 | 0.771 | 0.318 | -0.125 |
| DASS_16 | 0.960 | 0.791 | 0.085 | -0.085 |
| DASS_09 | 0.837 | 0.534 | 0.068 | -0.068 |
| DASS_08 | 1.347 | 0.955 | 0.058 | 0.058 |
| DASS_10 | 0.991 | 0.807 | 0.056 | -0.056 |
| DASS_05 | 0.816 | 0.713 | 0.051 | -0.051 |
| DASS_14 | 0.806 | 0.707 | 0.051 | 0.051 |
| DASS_07 | 0.942 | 0.942 | 0.043 | 0.043 |
| DASS_19 | 0.918 | 0.791 | 0.042 | -0.042 |
| DASS_11 | 1.273 | 1.203 | 0.035 | -0.035 |
| DASS_17 | 1.558 | 0.921 | 0.056 | 0.035 |
| DASS_06 | 1.039 | 0.984 | 0.028 | -0.028 |
| DASS_21 | 1.076 | 0.962 | 0.023 | -0.023 |
| DASS_03 | 1.033 | 1.033 | 0.018 | 0.018 |
| OEA | 0.710 | 0.703 | 0.022 | 0.015 |
| DASS_01 | 0.744 | 0.721 | 0.012 | -0.012 |
| SEA | 1.117 | 0.983 | 0.146 | 0.011 |
| DASS_20 | 1.142 | 1.120 | 0.011 | -0.011 |
| DASS_02 | 0.629 | 0.587 | 0 | 0 |
| DASS_04 | 1.173 | 1.138 | 0 | 0 |
| DASS_12 | 1.009 | 1.009 | 0 | 0 |
| DASS_13 | 1.184 | 1.184 | 0 | 0 |
| DASS_15 | 1.173 | 1.163 | 0 | 0 |

Supplementary Table S3. Centrality and bridge centrality indices of emotional intelligence dimensions and DASS-21 symptom nodes

***Note.*** The table presents strength, expected influence, bridge strength, and bridge expected influence for each node in the network. Nodes are ordered by the absolute value of bridge expected influence. Higher strength and expected influence values indicate greater importance of a node within the overall network. Higher absolute bridge expected influence values indicate stronger cross-community connections between emotional intelligence dimensions and DASS-21 symptom nodes. SEA = self-emotion appraisal; OEA = others’ emotion appraisal; UOE = use of emotion; ROE = regulation of emotion; DASS_01-DASS_21 = individual symptom nodes from the Depression Anxiety Stress Scales-21.

Supplementary Table S4. Nonzero cross-community edge weights between emotional intelligence dimensions and DASS-21 symptom nodes

| EI_Dimension | DASS_Item | Edge_Weight |
| --- | --- | --- |
| ROE | DASS_18 | -0.186 |
| UOE | DASS_16 | -0.085 |
| ROE | DASS_09 | -0.068 |
| SEA | DASS_08 | 0.058 |
| UOE | DASS_10 | -0.056 |
| ROE | DASS_17 | 0.046 |
| UOE | DASS_07 | 0.043 |
| SEA | DASS_11 | -0.035 |
| UOE | DASS_19 | -0.034 |
| UOE | DASS_14 | 0.030 |
| UOE | DASS_05 | -0.028 |
| ROE | DASS_06 | -0.028 |
| ROE | DASS_05 | -0.024 |
| UOE | DASS_18 | 0.023 |
| SEA | DASS_14 | 0.021 |
| UOE | DASS_21 | -0.019 |
| OEA | DASS_03 | 0.018 |
| SEA | DASS_01 | -0.012 |
| SEA | DASS_20 | -0.011 |
| SEA | DASS_17 | -0.010 |
| ROE | DASS_19 | -0.008 |
| OEA | DASS_21 | -0.003 |
| OEA | DASS_14 | <0.001 |

***Note.*** Only nonzero cross-community edges retained in the EBICglasso network are presented. Edges with a weight of 0 were omitted because no direct cross-community association was retained after regularization. Edges are ordered by the absolute value of the edge weight in descending order. Negative values indicate inverse associations, and positive values indicate positive associations. The complete set of edge weights, including zeros, is available in the partial correlation matrix shown in Supplementary Table S2.

Supplementary Table S5. Comparison of centrality and bridge centrality indices across the unadjusted, work-experience-adjusted, and age-adjusted networks

| Node | Strength |  |  | EI |  |  | BEI |  |  |
| --- | --- | --- | --- | --- | --- | --- | --- | --- | --- |
|  | Unadjusted | Adjusted for work experience | Adjusted for age | Unadjusted | Adjusted for work experience | Adjusted for age | Unadjusted | Adjusted for work experience | Adjusted for age |
| SEA | 1.117 | 0.994 | 0.995 | 0.983 | 0.953 | 0.954 | 0.011 | -0.008 | −0.008 |
| OEA | 0.710 | 0.682 | 0.682 | 0.703 | 0.682 | 0.682 | 0.015 | 0 | 0 |
| UOE | 1.214 | 1.071 | 1.071 | 0.771 | 0.701 | 0.701 | -0.125 | -0.185 | −0.185 |
| ROE | 0.987 | 0.912 | 0.914 | 0.359 | 0.333 | 0.332 | -0.268 | -0.290 | −0.291 |
| DASS_01 | 0.744 | 0.744 | 0.741 | 0.721 | 0.744 | 0.741 | -0.012 | 0 | 0 |
| DASS_02 | 0.629 | 0.621 | 0.619 | 0.587 | 0.621 | 0.619 | 0 | 0 | 0 |
| DASS_03 | 1.033 | 1.020 | 1.020 | 1.033 | 1.020 | 1.020 | 0.018 | 0 | 0 |
| DASS_04 | 1.173 | 1.072 | 1.074 | 1.138 | 1.072 | 1.074 | 0 | 0 | 0 |
| DASS_05 | 0.816 | 0.763 | 0.763 | 0.713 | 0.687 | 0.684 | -0.051 | -0.038 | −0.040 |
| DASS_06 | 1.039 | 1.002 | 1.002 | 0.984 | 0.950 | 0.947 | -0.028 | -0.026 | −0.028 |
| DASS_07 | 0.942 | 0.816 | 0.816 | 0.942 | 0.816 | 0.816 | 0.043 | 0 | 0 |
| DASS_08 | 1.347 | 0.966 | 0.967 | 0.955 | 0.908 | 0.910 | 0.058 | 0.012 | 0.013 |
| DASS_09 | 0.837 | 0.697 | 0.697 | 0.534 | 0.569 | 0.569 | -0.068 | -0.064 | −0.064 |
| DASS_10 | 0.991 | 0.953 | 0.950 | 0.807 | 0.828 | 0.825 | -0.056 | -0.062 | −0.063 |
| DASS_11 | 1.273 | 1.208 | 1.207 | 1.203 | 1.168 | 1.167 | -0.035 | -0.020 | −0.020 |
| DASS_12 | 1.009 | 1.063 | 1.064 | 1.009 | 1.063 | 1.064 | 0 | 0 | 0 |
| DASS_13 | 1.184 | 1.166 | 1.167 | 1.184 | 1.166 | 1.167 | 0 | 0 | 0 |
| DASS_14 | 0.806 | 0.646 | 0.643 | 0.707 | 0.646 | 0.643 | 0.051 | 0 | 0 |
| DASS_15 | 1.173 | 1.115 | 1.114 | 1.163 | 1.115 | 1.114 | 0 | 0 | 0 |
| DASS_16 | 0.960 | 0.922 | 0.922 | 0.791 | 0.780 | 0.781 | -0.085 | -0.071 | −0.071 |
| DASS_17 | 1.558 | 0.751 | 0.750 | 0.921 | 0.693 | 0.692 | 0.035 | 0 | 0 |
| DASS_18 | 1.007 | 0.918 | 0.918 | 0.635 | 0.598 | 0.596 | -0.163 | -0.160 | −0.161 |
| DASS_19 | 0.918 | 0.866 | 0.868 | 0.791 | 0.805 | 0.815 | -0.042 | -0.031 | −0.027 |
| DASS_20 | 1.142 | 1.114 | 1.115 | 1.120 | 1.103 | 1.104 | -0.011 | -0.005 | −0.005 |
| DASS_21 | 1.076 | 0.909 | 0.908 | 0.962 | 0.873 | 0.871 | -0.023 | -0.018 | −0.019 |

***Note.*** The work-experience-adjusted and age-adjusted networks were estimated separately using a residual network approach. The corresponding columns present values obtained after controlling for years of work experience or age, respectively. Strength and expected influence indicate node centrality within the overall network. Higher absolute bridge expected influence values indicate stronger cross-community connections between emotional intelligence dimensions and DASS-21 symptom nodes. EI = expected influence; BEI = bridge expected influence; SEA = self-emotion appraisal; OEA = others’ emotion appraisal; UOE = use of emotion; ROE = regulation of emotion; DASS_01–DASS_21 = individual symptom nodes from the Depression Anxiety Stress Scales-21.

Supplementary Table S6. Top 10 cross-community edge weights in the unadjusted, work-experience-adjusted, and age-adjusted networks

| Unadjusted network | | |  | Work-experience-adjusted network | | |  | Age-adjusted network | | |
| --- | --- | --- | --- | --- | --- | --- | --- | --- | --- | --- |
| EI_**Dimension** | DASS_**Item** | E**dge_Weight** |  | EI_**Dimension** | DASS_**Item** | E**dge_Weight** |  | EI_**Dimension** | DASS_**Item** | E**dge_Weight** |
| ROE | DASS_18 | -0.186 |  | ROE | DASS_18 | -0.160 |  | ROE | DASS_18 | -0.161 |
| UOE | DASS_16 | -0.085 |  | UOE | DASS_16 | -0.071 |  | UOE | DASS_16 | -0.071 |
| ROE | DASS_09 | -0.068 |  | ROE | DASS_09 | -0.064 |  | ROE | DASS_09 | -0.064 |
| SEA | DASS_08 | 0.058 |  | UOE | DASS_10 | -0.062 |  | UOE | DASS_10 | -0.062 |
| UOE | DASS_10 | -0.056 |  | ROE | DASS_05 | -0.027 |  | ROE | DASS_05 | -0.028 |
| ROE | DASS_17 | 0.046 |  | ROE | DASS_06 | -0.026 |  | ROE | DASS_06 | -0.028 |
| UOE | DASS_07 | 0.043 |  | UOE | DASS_19 | -0.023 |  | UOE | DASS_19 | -0.022 |
| SEA | DASS_11 | -0.035 |  | SEA | DASS_11 | -0.020 |  | SEA | DASS_11 | -0.020 |
| UOE | DASS_19 | -0.034 |  | UOE | DASS_21 | -0.018 |  | UOE | DASS_21 | -0.019 |
| UOE | DASS_14 | 0.030 |  | SEA | DASS_08 | 0.012 |  | SEA | DASS_08 | 0.013 |

***Note.*** The left, middle, and right panels present the top 10 nonzero cross-community edges in the unadjusted, work-experience-adjusted, and age-adjusted networks, respectively. The work-experience-adjusted and age-adjusted networks were estimated separately. Only cross-community edges between emotional intelligence dimensions and DASS-21 symptom nodes are shown. Edges with a weight of 0 were omitted because no direct cross-community association was retained after regularization. Edges are ordered by the absolute value of edge weight in descending order within each network. Negative values indicate inverse associations, whereas positive values indicate positive associations. EI_dimension = emotional intelligence dimension; DASS_item = DASS-21 symptom node.

Supplementary Figure S1. **Flow diagram of participant recruitment, eligibility assessment, and data screening**

***Note.*** A total of 756 respondents initiated the questionnaire. Of these, 709 completed it and 47 did not, yielding a completion rate of 93.8%. Five respondents who did not hold a valid nursing license were excluded, resulting in a final analytic sample of 704 clinical nurses. No duplicate responses, implausible values, or missing data in the study variables were identified. The total number of nurses invited to participate and the number who declined were not recorded; therefore, a response rate could not be calculated.

****Supplementary Figure S2.**** Heatmap of Pearson correlations between emotional intelligence dimensions and DASS-21 subscales

***Note.*** The figure presents Pearson correlation coefficients among the four dimensions of emotional intelligence and the three DASS-21 subscales. Red indicates positive correlations and blue indicates negative correlations. Darker colours represent stronger associations. SEA = self-emotion appraisal; OEA = others’ emotion appraisal; UOE = use of emotion; ROE = regulation of emotion; DASS-21 = Depression Anxiety Stress Scales.

Supplementary Figure S3. Bootstrapped difference tests for edge weights

*Note.* Gray boxes indicate no significant difference between two edge weights, whereas black boxes indicate a significant difference. On the diagonal, blue boxes indicate positive edge weights and red boxes indicate negative edge weights. SEA = Self-Emotion Appraisal; OEA = Others’ Emotion Appraisal; UOE = Use of Emotion; ROE = Regulation of Emotion. D01–D21 denote DASS_01–DASS_21, respectively. DASS_01: Difficulty winding down; DASS_02: Dry mouth; DASS_03: Lack of positive feelings; DASS_04: Breathing difficulty; DASS_05: Lack of initiative; DASS_06: Overreactivity; DASS_07: Trembling; DASS_08: Nervous energy; DASS_09: Panic-related worry; DASS_10: Nothing to look forward to; DASS_11: Agitation; DASS_12: Difficulty relaxing; DASS_13: Low mood; DASS_14: Intolerance of interruptions; DASS_15: Feeling close to panic; DASS_16: Lack of enthusiasm; DASS_17: Low self-worth; DASS_18: Irritability; DASS_19: Awareness of heartbeat; DASS_20: Unexplained fear; DASS_21: Meaninglessness.

Supplementary Figure S4. Bootstrapped difference tests for node expected influence (EI)

*Note.* Gray boxes represent no significant difference between nodes; while black boxes represent significant difference. White boxes on the diagonal represent the values of node EI. SEA: Self-Emotion Appraisal; OEA: Others' Emotion Appraisal; UOE: Use of Emotion; ROE: Regulation of Emotion; DASS_01: Difficulty winding down; DASS_02: Dry mouth; DASS_03: Lack of positive feelings; DASS_04: Breathing difficulty; DASS_05: Lack of initiative; DASS_06: Overreactivity; DASS_07: Trembling; DASS_08: Nervous energy; DASS_09: Panic-related worry; DASS_10: Nothing to look forward to; DASS_11: Agitation; DASS_12: Difficulty relaxing; DASS_13: Low mood; DASS_14: Intolerance of interruptions; DASS_15: Feeling close to panic; DASS_16: Lack of enthusiasm; DASS_17: Low self-worth; DASS_18: Irritability; DASS_19: Awareness of heartbeat; DASS_20: Unexplained fear; DASS_21: Meaninglessness.

Supplementary Figure S5. Bootstrapped difference tests for bridge expected influence (BEI)

*Note.* Gray boxes represent no significant difference between nodes; while black boxes represent significant difference. White boxes on the diagonal represent the values of node BEI. SEA: Self-Emotion Appraisal; OEA: Others' Emotion Appraisal; UOE: Use of Emotion; ROE: Regulation of Emotion; DASS_01: Difficulty winding down; DASS_02: Dry mouth; DASS_03: Lack of positive feelings; DASS_04: Breathing difficulty; DASS_05: Lack of initiative; DASS_06: Overreactivity; DASS_07: Trembling; DASS_08: Nervous energy; DASS_09: Panic-related worry; DASS_10: Nothing to look forward to; DASS_11: Agitation; DASS_12: Difficulty relaxing; DASS_13: Low mood; DASS_14: Intolerance of interruptions; DASS_15: Feeling close to panic; DASS_16: Lack of enthusiasm; DASS_17: Low self-worth; DASS_18: Irritability; DASS_19: Awareness of heartbeat; DASS_20: Unexplained fear; DASS_21: Meaninglessness.

Supplementary Figure S6. Network structures estimated after separate adjustment for years of work experience and age

*Note.* The left panel presents the network adjusted for years of work experience, and the right panel presents the network adjusted for age. The two networks were estimated separately using a residual network approach. For each network, the WLEIS dimensions and DASS-21 items were regressed on the corresponding covariate, and the resulting residuals were used to estimate the EBICglasso network. Nodes represent the four emotional intelligence dimensions and 21 DASS-21 symptom items. Blue edges indicate positive conditional associations, whereas red edges indicate negative conditional associations. Edge thickness represents the absolute magnitude of the association.

Supplementary Figure S7. Case-dropping bootstrap results for centrality indices and bridge expected influence in networks separately adjusted for years of work experience and age

*Note.* The upper panel presents results for the network adjusted for years of work experience, and the lower panel presents results for the network adjusted for age. The lines show the average correlations between centrality estimates obtained from case-dropping subsamples and the corresponding full-sample estimates.

Supplementary Figure S8. Comparison of network structures between nurses with and without patient aggression experience

*Note.* The left panel shows the network of nurses without patient aggression experience, and the right panel shows the network of nurses with patient aggression experience. The color of each node signifies its community belonging. Blue edges indicate positive associations, and red edges indicate negative associations between nodes. Edge thickness reflects the absolute magnitude of the regularized partial correlation.
